# Short-term audiological outcomes of the mCLIP ARC ball joint prosthesis– a retrospective multicenter study

**DOI:** 10.1007/s00405-025-09475-w

**Published:** 2025-05-29

**Authors:** Dirk Beutner, N. Bevis, S. Arndt, C. Offergeld, T. Lenarz, S. Busch, W. Wolfram, L. Niederwanger, B. Loader, F. Windisch, C. Arnoldner, D. Riss, E. Schimanski, P. M. Zwittag, N. Rubicz, P. H. Skarżyński, Ł. Plichta, G. Sprinzl, A. Magele, L. Taha, J. Hornung

**Affiliations:** 1 Department of Otorhinolaryngology, Head and Neck Surgery, University Medical Center Goettingen, Robert-Koch-Straße 40, 37075 Goettingen, Germany; 2 Department of Otorhinolaryngology-Head and Neck Surgery, Faculty of Medicine, Medical Center-University of Freiburg, University of Freiburg, 79106 Freiburg, Germany; 3 Department of Otolaryngology, Hannover Medical School, 30625 Hannover, Germany; 4 Cluster of Excellence Hearing4all, Medical University Hannover, 30625 Hannover, Germany; 5 Department of Otorhinolaryngology–Klinikum Wels-Grieskirchen, Wels, 4600 Austria; 6 Department of Otorhinolaryngology, Head and Neck Surgery, Klinik Landstraße, Wiener Gesundheitsverbund, Vienna, 1030 Austria; 7 Sigmund Freud Private University, Vienna, 1020 Austria; 8 Department of Otorhinolaryngology, Medical University of Vienna, Vienna, 1090 Austria; 9 Zentrum Fuer Mittelohrchirurgie (Centre for Middle Ear Surgery), ENT Practice, 44536 Luenen, Germany; 10 Department of Otorhinolaryngology, Head and Neck Surgery, Kepler University Hospital GmbH, Linz, 4020 Austria; 11 Medical Faculty, Johannes Kepler University Linz, Altenbergerstrasse 69, Linz, 4040 Austria; 12 Department of Teleaudiology and Screening, Institute of Physiology and Pathology of Hearing, Warsaw, Kajetany, Poland; 13 Institute of Sensory Organs, Kajetany, Poland; 14 Heart Failure and Cardiac Rehabilitation Department, Faculty of Dental Medicine, Medical University of Warsaw, Warszawa, Poland; 15 Oto-Rhino-Laryngology Surgery Clinic, Institute of Physiology and Pathology of Hearing, Warsaw, Kajetany, Poland; 16 Department of Otorhinolaryngology, Head & Neck Surgery, University Clinic St. Poelten, St. Pölten, 3100 Austria; 17 Department of Otorhinolaryngology, Head & Neck Surgery, University of Erlangen- Nuremberg, 91054 Erlangen, Germany

**Keywords:** Middle ear prosthesis, Partial ossicular replacement prosthesis, Ball joint

## Abstract

**Purpose:**

This European multicentric, retrospective study aimed to analyze the short-term safety and effectiveness of the mCLIP ARC Ball Joint Prosthesis.

**Methods:**

Type III Tympanoplasty with implantation of a mCLIP-ARC Partial Prosthesis was performed. During patients’ follow-up, ear microscopic evaluation and pure-tone audiometry were used for analysis. The post-operative pure-tone average of the frequencies 0.5, 1, 2 and 3 kHz (PTA4) were used for calculation of the air bone gap (ABG). Adverse events (AE) were evaluated during follow-up.

**Results:**

62 patients underwent implantation of the mCLIP-ARC Partial Prosthesis. 55 complete data sets were available for audiological examination. The mean follow-up time was 58 days. In two cases adverse events were recorded. The post-operative bone conduction (BC) PTA4 thresholds were stable in 96.4% of cases. The mean postoperative PTA-4 air-bone gap (ABG) was 16.5 ± 9.2 dB.

**Conclusion:**

Implantation of the mCLIP ARC partial prosthesis proves to be safe and reliable. Clinical data show satisfactory audiological results and demonstrate the effectiveness of the new middle ear prosthesis with a balanced ball joint.

## Introduction

Hearing is essential for communication and social life. Restoring hearing ability in the diseased middle ear by tympanoplasty is the clinical standard since its introduction by Wullstein in the last century [1]. Passive middle ear implants are key elements for reconstruction of the middle ear [2]. In partial ossicular reconstruction, the physiological function of the ossicular chain is lost due to interposition of rigid middle ear prostheses. In this case, an acoustic bypass is created that avoids the incudomalleolar joint, which plays a crucial role in adjusting and regulating atmospheric pressure changes [3]. Standard partial ossicular replacement prosthesis (PORP) can give excellent acoustic results, however due to its rigid design it lacks the ability to adapt to pressure changes. This in conjunction with postoperative scarring can lead to dislocation of the PORP and are a major factor in poor postoperative hearing results. Furthermore, pressure points in contact areas between the tympanic membrane and the rigid implant can eventually lead to the extrusion of the PORP. As a result, research has focused on middle ear implants that replicate the ossicular joints and can adjust to the conical shape of the tympanic membrane [4, 5]. To address the limitations of earlier prostheses featuring a ball joint between the prosthesis shaft and plate, the mCLIP-ARC partial ossicular prosthesis was developed [6, 7]. In the following, we evaluate the safety and effectiveness of the mclip-ARC in a multicenter study.

## Materials and methods

**Fig. 1:**
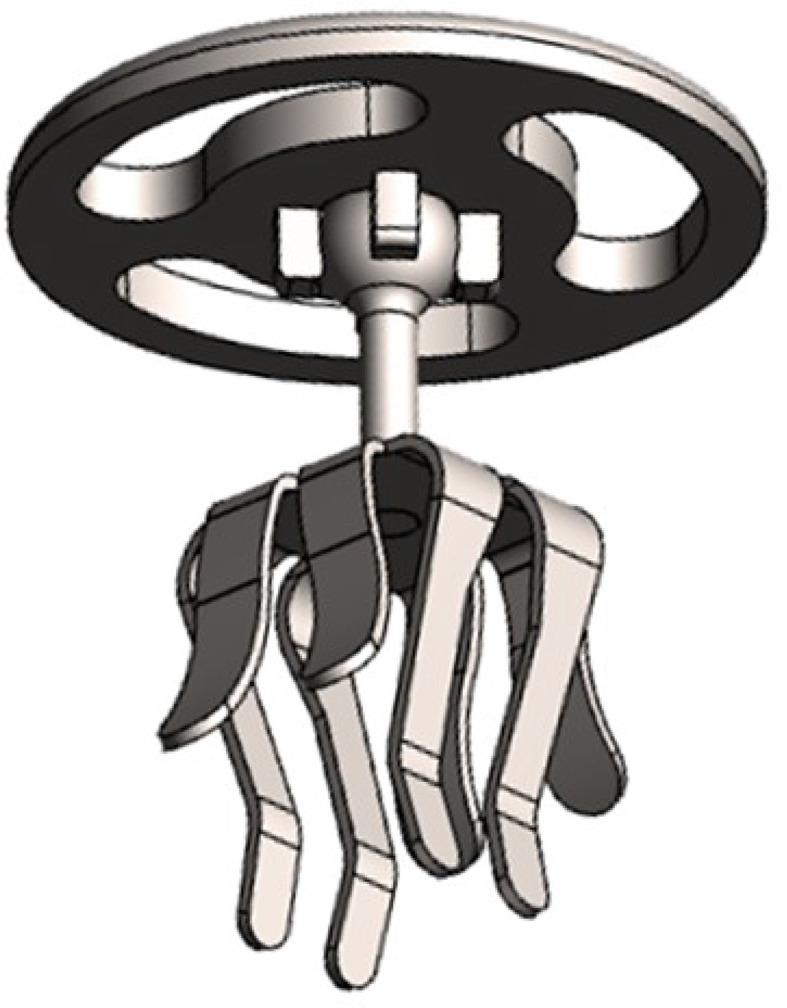
3D rendering of the novel stapes prosthesis. Measurements in mm

**Fig. 2:**
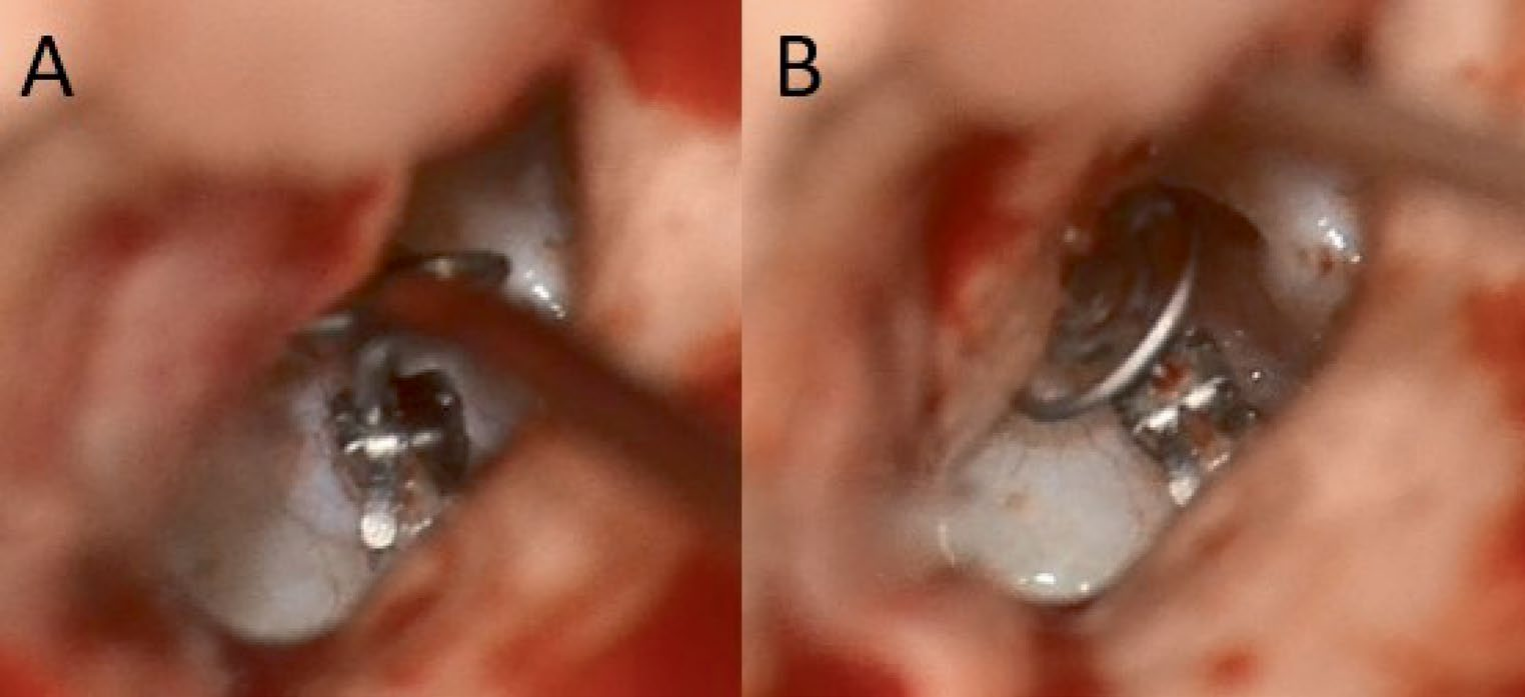
Intraoperative view of the mCLIP ARC partial prosthesis. during the clipping process (**A**), the prosthesis headplate remains deflected to grant the surgeon a full overview of the anatomical structures. After securing the prosthesis to the stapes suprastructure, the headplate can be deflected (**B**) to achieve an orthogonal position to the plane of the tympanic membrane

The mCLIP ARC titanium ball joint prosthesis (MED-EL, Innsbruck, Austria) is available in several lengths (0.75–3.50 mm in increments of 0.25–0.5 mm). Figure 1 illustrates its design. The prosthesis is constructed from titanium and employs a reliable clip design to attach it to the stapes head [8]. A ball joint linking the headplate and shaft enables the headplate to rotate and tilt in relation to the shaft. (Figures 1 and 2)

The study was conducted in a retrospective, multicenter setting and implantations took place between January 2021 and December 2022. The audiometric assessments took place preoperatively and at one short-term follow-up (57.5 ± 50.6 days). In total, 62 cases were implanted, and 55 cases returned for postoperative follow-up. 36 patients suffered from cholesteatoma. Information on adverse events was collected. The pure-tone average (PTA-4) at 0.5, 1, 2, and 3 kHz was calculated according to the guidelines of the American Academy of Otolaryngology–Head and Neck Surgery [9].

Statistical analyses were performed using Origin (Ver. 10.5.126) and Microsoft Excel. Results were presented as mean values ± standard deviation. A one-way repeated measures ANOVA test was used to assess differences in bone-conduction measurements across time-points. The study was carried out in 11 clinics in Germany, Austria, and Poland. They were done in conformity with the Declaration of Helsinki and were approved by the corresponding ethics committees (Goettingen: 1/9/20; Hannover: 9456_BO_S_2020; Erlangen: 456_20 Bc; Lünen: 2020-829-b-S; Freiburg: 22-1142-retro; Linz: 1257/2022; Wels: 1257/2022; Gesundheitsverbund, Klinik Landstraße: EK_23_005_XX; AKH Vienna: 2296/2021; Sankt Poelten: GS1-EK-4/777–2022; Warsaw: Oświadczenie nr. 10/2023r). This study was registered at ClinicalTrials.gov (NCT05565339).

## Results

In total, 62 implantations of the ball joint prosthesis were performed between September 2021 and December 2022. Of the 62 cases, 55 were available for audiometric evaluation since 7 patients did not report to the audiological follow-up. Table 1 gives details of the patients, in which 49.1% were female and 50.9% male. Patients were implanted at an average age of 46.5 (± 19.5) years, and the average follow-up time was 57.5 ± 50.6 days (Table 1).

**Table 1:** Patient characteristics

*n*					55
Sex (male/female)					28 / 27
Age at surgery					46.5 ± 19.5
Cholesteatoma surgery (n)					36
Early follow up in days (mean + SD)					57.5 ± 50.6

**Fig. 3:**
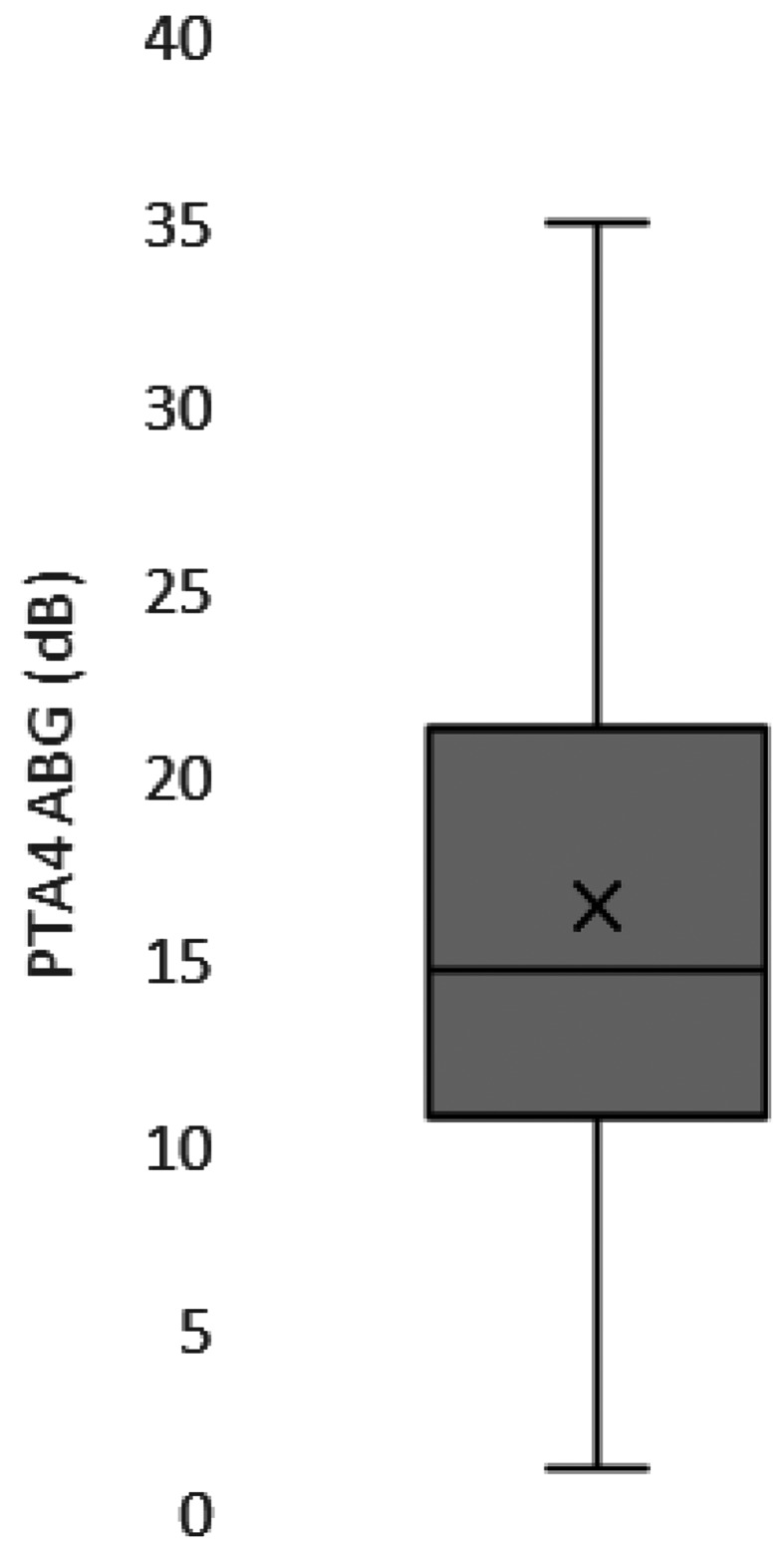
The average PTA-4 ABG at 57.5 ± 50.6 days after surgery was 16.5 ± 9.2 dB (*n* = 55). Boxplots show upper and lower limits (whiskers), median (horizontal line), and mean (cross)

**Fig. 4:**
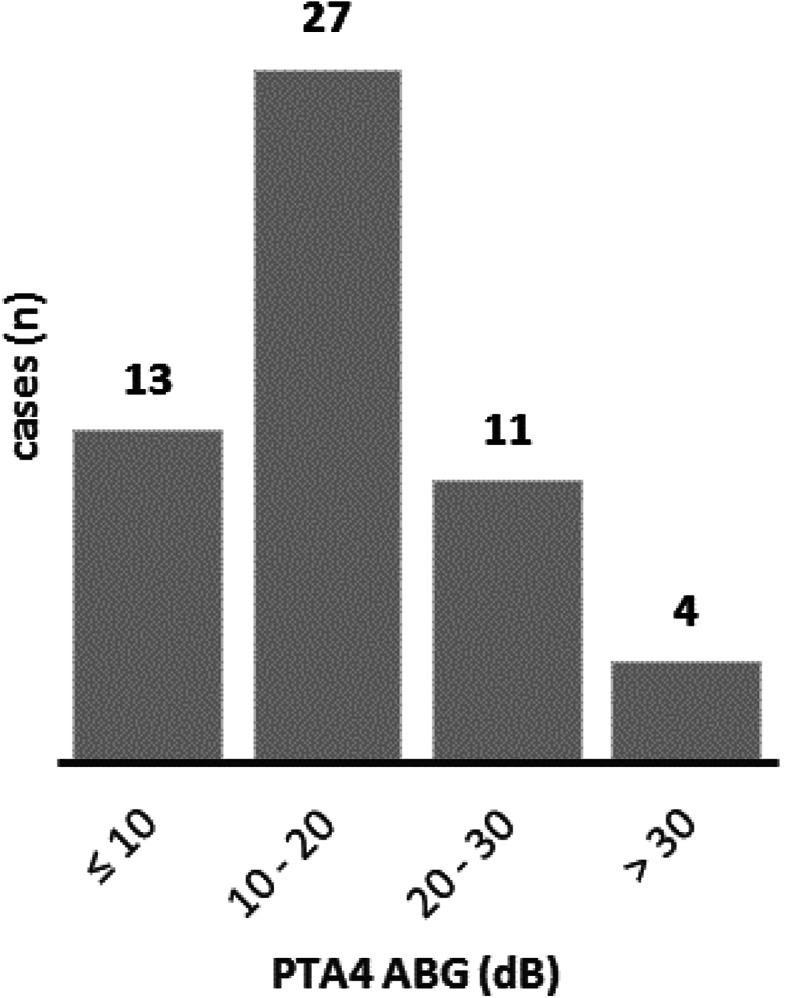
Histogram of postoperative PTA-4 ABG (*n* = 55). In 13 patients (23.6%), the postoperative PTA-4 ABG was within 10 dB and in 40 patients (72.7%) within 20 dB

Of the 55 patients who reported for follow-up, pure-tone audiometry showed that the mean postoperative PTA-4 air-bone gap (ABG) was 16.5 ± 9.2 dB. In 13 patients (23.6%), the postoperative PTA-4 ABG was within 10 dB and in 40 patients (72.7%) within 20 dB. The postoperative PTA-4 thresholds for bone conduction (BC) were stable in 96.4% of cases (53 of 55). The average PTA-4 BC threshold was 18.2 ± 13.8 dB preoperatively and 15.9 ± 13.6 dB postoperatively (Figs. 3 and 4).

**Fig. 5:**
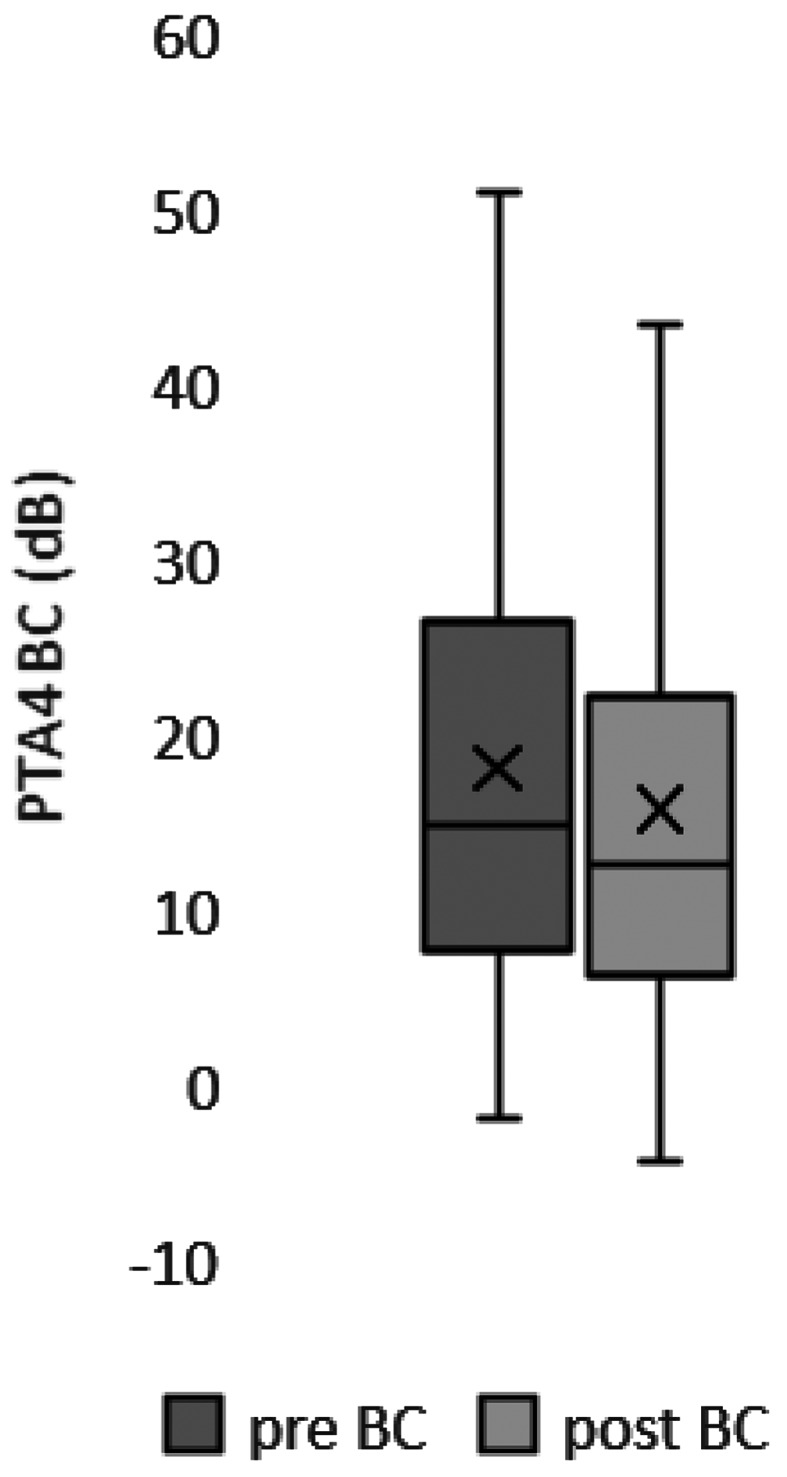
The average PTA-4 BC threshold was 18.2 ± 13.8 dB preoperatively and 15.9 ± 13.6 dB postoperatively (*p* = 0.51) and was stable in 96.4% of patients. Boxplots show upper and lower limits (whiskers), median (horizontal line), and mean (cross)

Statistical analysis on the pre- and postoperative audiological measurements was performed by one-way ANOVA with Tukey’s multiple comparison. There was no significant change in PTA-4 bone conduction thresholds pre- to postoperatively (*p* = 0.51). There was no significant difference between the mean values of the pre- and postoperative bone conduction hearing thresholds (Fig. 5).

In two cases (2/62 = 3.2%), three adverse events were reported. One patient experienced a recurrent tympanic membrane perforation, which was resolved by a revision surgery. Another patient showed a small tympanic membrane perforation and taste disturbance. No revision surgery was needed. No patient complained of tinnitus or facial nerve paralysis. No device-related adverse events were reported.

## Discussion

The aim of this multicenter study was to investigate the safety and short-term audiological outcomes of the mCLIP ARC partial prosthesis.

In partial ossicular reconstruction, a firm and stable anchorage of the PORP to the tympanic membrane as well as stapes suprastructure is crucial for an adequate hearing outcome of implanted patients. To achieve a standardized coupling to the stapes suprastructure, the titanium clip prosthesis was developed by Hüttenbrink et al. [8]. The innovative Dresden-clip has proven its safety and effectiveness in various designs for passive and active middle ear implants [2, 4, 10]. Furthermore, long-term experience demonstrate the advantages of the self-retaining design that is associated with a low risk of dislocation, easy to remove during revision surgeries and long-term hearing effectiveness [11]. Recent developments in middle ear implants incorporate a ball joint to mimic the function of the incudomalleolar joint with good clinical results [4, 12]. Further improving the ball joint concept, the newly developed mCLIP ARC is equipped with a centered ball joint between prosthesis headplate and shaft that provides a more balanced adaptation to individual anatomical needs and reduces the risk of complete deflection of the headplate over an asymmetrical design. Preclinical data from temporal bone experiments showed the excellent acoustic performance under laboratory conditions and the general usability of the ball joint under physiological loads and forces [6]. The newly developed mCLIP ARC [6, 7] was CE-certified and released to the market in 2020. First clinical experiences demonstrated the general usability of the prosthesis in a monocentric study [7]. The application and placement is straightforward and the angle between prosthesis shaft and headplate was always between 45 and 90 degree, indicating the need and the benefit of the microjoint to adapt to the individual anatomical situations of the tympanic membrane. Moreover, deflection of the headplate allows the surgeon a full overview of the oval niche that reduces the risk of prosthesis malposition or traumatic damage to the stapes or annular ligament. Another benefit of the flexible headplate is that it prevents large atmospheric pressure fluctuations from being fully transmitted to the inner ear. The microjoint absorbs applied forces, contrary to rigid prostheses that cannot adjust its headplate. It is tempting to speculate that this characteristic of the mCLIP ARC is advantageous for superior hearing results as also tension applied to the annular ligament is minimized [13]. Moreover, the continuous, individualized adjustment of the mobile headplate helps minimize pressure points in the contact areas, thereby lowering the risk of protrusion, particularly in high-risk ears [14]. Therefore, we expect a positive impact of the mCLIP ARC on revision rates in long-term studies.

Bevis et al. reported that the mCLIP ARC is safe and effective with the postoperative PTA-4 ABG significantly decreasing from a mean of 24.5 (± 11) dB to 17.4 (± 7.9) dB, while the Dresden partial prosthesis showed a significant decrease of PTA4-ABG from a mean of 25.0 (± 10.9) dB to 20.1 (± 10.9) dB at short-term follow-up. This comparison showed a slightly better but not significant outcome of the ball-joint prosthesis [7]. This is in line and comparable to the here reported multicentric data where the mean PTA-4 ABG was 16.5 dB and a good match with previously published multicentric data on the rigid mCLIP prosthesis with a mean PTA-4 ABG of 16.3 dB [15].

The audiological outcome compares favorably with those from other studies [16, 17]. However, care must be taken regarding the interpretation of these favorable results, as the final hearing outcome is influenced by many, also prosthesis independent factors such as patient population, underlying disease, surgeons experience, status of the mucosa and aeration of the middle ear [2, 18, 19, 20].

The limitations of this study are the lack of a control group, the short follow-up period, and the absence of preoperative data on the ABG. The latter make it difficult to assess the improvements due to prosthesis implantation. Despite these initial findings, further research with a larger patient group and extended follow-up is required to validate the hypothesis that the ball joint absorbs micromovements at the tympanic membrane, reduces strain on the annular ligament, and consequently leads to more stable audiological results over time, along with a lower incidence of revision surgeries due to prosthesis migration.

Short-term follow-up showed satisfying audiological results and confirmed the safety of the implant. Nevertheless, it is tempting to speculate that this innovation will lead to better audiological results and fewer revision surgeries due to dislocation or extrusion of the implant in the long term.

The multicenter data provides evidence that the mCLIP ARC represents a significant advancement in the development of a functional passive middle ear prosthesis, capable of restoring both key middle ear functions: sound conduction and cochlear protection from large atmospheric pressure fluctuations.

## Conclusion

The implantation of the mCLIP ARC middle ear prosthesis with a centered ball joint is safe and leads to excellent short-term audiological results.
